# “I would walk through fire to get this vaccine”: a mixed-methods study examining attitudes and perceptions of a gonorrhoea vaccine programme among UK sexual health service users

**DOI:** 10.1136/bmjph-2025-003819

**Published:** 2026-03-27

**Authors:** Charlie Firth, Sophia Wilkinson, Sam Martin, Samantha Vanderslott, Andrew J Pollard, Katrina M Pollock

**Affiliations:** 1 Oxford Vaccine Group, Department of Paediatrics, University of Oxford, Oxford, UK; 2 NIHR Oxford Biomedical Research Centre, Oxford, UK; 3 Pandemic Sciences Institute, Nuffield Department of Medicine, University of Oxford, Oxford, UK; 4 Oxfordshire Sexual Health Service, Oxford University Hospitals NHS Foundation Trust, Oxford, UK

**Keywords:** Vaccination, Qualitative Research, Community-Based Participatory Research, Public Health, Sexual Health

## Abstract

**Introduction:**

Antimicrobial-resistant gonorrhoea poses a significant global public health threat. In response, the UK launched the world’s first gonorrhoea vaccination programme using a 4CMenB vaccine, targeting individuals at high risk of bacterial sexually transmitted infections (STIs). Understanding attitudes and acceptability among sexual health service users is essential to inform successful implementation.

**Methods:**

The NAVIGATE study employed a mixed-methods design, combining an online survey (n=395) with semi-structured interviews (n=15) among UK adults who had previously accessed sexual health services. The survey included demographic questions, the validated Vaccination Attitudes Examination scale and items assessing the acceptability of gonorrhoea vaccine. Interviews explored deeper perceptions of vaccination, trust and delivery preferences. Quantitative data were analysed descriptively and qualitative data thematically using Braun and Clarke’s framework.

**Results:**

Participants showed high general vaccine confidence, with 92.2% strongly supporting a gonorrhoea vaccine programme. Acceptance was driven by concerns about antimicrobial resistance and trust in UK regulatory systems. Stigma and structural barriers, such as fear of judgement, cost and inconvenient delivery settings, were key factors influencing access. Participants favoured delivery through sexual health clinics and LGBTQ+ venues and strongly endorsed peer-led outreach. Partial efficacy of the proposed vaccine was accepted when transparently communicated. Concerns over personal costs emerged as potentially worsening inequalities.

**Conclusions:**

While overall acceptability was high, successful uptake requires delivery that is trusted, stigma-free, accessible and clearly communicated. Policy recommendations include ensuring free access, leveraging peer-led campaigns and delivering vaccination in low-barrier settings. Monitoring by demographic group is essential to prevent inequities. These insights are crucial as the UK’s pioneering programme generates global evidence for future STI vaccine policy.

**WHAT IS ALREADY KNOWN ON THIS TOPIC**

- Gonorrhoea is becoming increasingly resistant to antibiotics, and modelling suggests that vaccination could help reduce disease burden. However, public perceptions remain a critical factor influencing uptake. Previous research has shown that concerns about low vaccine efficacy, stigma associated with sexually transmitted infections (STIs) and mistrust of health institutions can limit the acceptability of STI vaccination programmes.

**WHAT THIS STUDY ADDS**

- This study demonstrates high acceptability of a gonorrhoea vaccination programme among UK sexual health service users, driven by concerns about antimicrobial resistance and strong trust in UK regulatory systems. It also identifies key implementation barriers. These include stigma in healthcare settings, cost concerns and delivery preferences. This study also highlights the importance of peer-led outreach and transparent communication about partial efficacy.

**HOW THIS STUDY MIGHT AFFECT RESEARCH, PRACTICE OR POLICY**

- These findings provide clear, actionable recommendations for delivering the gonorrhoea vaccine effectively and equitably. Policies should ensure free access, prioritise delivery through trusted and stigma-free settings such as sexual health clinics, and engage communities through peer-led campaigns. These insights will inform both national programme implementation and future international STI vaccine strategies.

## Introduction

 Gonorrhoea is an urgent global public health challenge. In the UK, diagnoses reached a record high of 85 370 in 2023,^1^ a burden that disproportionately affects gay, bisexual and other men who have sex with men (GBMSM).^2^ Rising levels of antimicrobial resistance (AMR) in *Neisseria gonorrhoea*, exemplified by the 14 ceftriaxone-resistant *N. gonorrhoeae* cases reported within the first 5 months of 2025, surpassing the total for all of 2024, further underscore the need for preventive approaches.^1^ In response, the UK Joint Committee on Vaccination and Immunisation (JCVI) recommended the targeted use of the 4CMenB vaccine, Bexsero, originally developed to prevent serogroup B meningococcal disease, as a preventive intervention against gonorrhoea.^3^

Genetic and phenotypic similarities exist between the two pathogens, *N. gonorrhoeae* and *N. meningitidis*, in their outer membrane vesicle (OMV). Given that this is contained in Bexsero, there is a theoretical likelihood of cross-protection through vaccination. A study of an earlier OMV containing vaccine, MeNZB, demonstrated incidental evidence of possible cross-protection against gonorrhoea in teenagers and young adults, with an effectiveness estimate of 31% (95% CI 21% to 39%).^4^ Other studies have given effectiveness estimates ranging from 33.2% to 59%.^5^,^9^ Recent randomised controlled trial (RCT) data from DOXYVAC, the first RCT of 4CMenB for the prevention of gonorrhoea, showed more modest effectiveness than observational studies.^10 11^

The UK Government has since accepted the JCVI’s recommendation, and a targeted vaccination programme for GBMSM and other individuals at high risk of bacterial sexually transmitted infections (STIs) who attend sexual health clinics commenced in August 2025.^12^ In the UK, sexual health services are free and confidential, delivered predominantly through specialist clinics (genitourinary medicine or sexual health clinics) and integrated within the National Health Service (NHS). As part of routine care, these services offer targeted vaccination against human papillomavirus (HPV) and hepatitis B for eligible groups and respond to outbreaks with timely vaccination campaigns, such as the targeted Mpox and hepatitis A immunisation programmes.^13^

Programme success is dependent on whether key target groups, such as GBMSM, will widely accept the 4CMenB vaccine, particularly in light of its relatively low predicted effectiveness.^14^ Acceptability has become an increasingly prominent concept in the evaluation of health interventions, reflecting recognition that an intervention’s effectiveness depends not only on clinical efficacy but also on how interventions are perceived and experienced by those who interact with them. In the context of vaccination, acceptability has been used to explore factors shaping public and caregiver responses to immunisation, including confidence in vaccine safety, perceptions of necessity and practical considerations such as access and convenience.^15^,^18^ While partially protective gonorrhoea vaccines offer promise, scientific uncertainties may hinder public confidence.

Notably, previous research on the HPV vaccine has demonstrated that perceived inefficacy of the vaccine can contribute to vaccine hesitancy and reduce vaccine acceptance,^19^ while the COVID-19 experience showed knowledge of vaccine effectiveness positively correlates with uptake.^20^ This highlights the importance of transparent communication about vaccine benefits. Recent analyses from a large, community-based survey of GBMSM in the UK found that while uptake of STI and viral hepatitis vaccinations such as HPV, hepatitis A and hepatitis B was relatively high (two-thirds), there remained considerable gaps, particularly among younger individuals, bisexual men and those with lower financial or social capital.^21^ GBMSM communities historically show high engagement with public health interventions,^22^ reflected in the rapid mpox vaccine uptake during 2022.^23 24^ However, STI-associated stigma may deter vaccination, and international experience raises equity concerns about access among vulnerable populations.^25^

A study in the USA^16^ of 4951 MSM found that 83.5% would be willing to receive a gonorrhoea vaccine, representing one of the few empirical studies to directly assess the acceptability of a gonorrhoea vaccine. Evidence beyond this is limited and fragmented, with only a small number of additional studies conducted in specific populations, such as incarcerated women.^26^ Furthermore, repurposing a 4CMenB vaccine may introduce unique acceptance barriers. A study in Japan examining willingness to receive the smallpox vaccine for protection against Mpox found low intention to accept the vaccine,^27^ highlighting the nuances in vaccine acceptability. Reflecting these gaps, experts have explicitly called for contemporary, context-specific research on gonorrhoea vaccine acceptability to inform implementation strategies.^28^ Given the scientific uncertainty, potential health inequities and public trust complexities, a mixed-methods study was conducted to examine the acceptability and perceptions of a gonorrhoea vaccine programme among UK sexual health service users in the UK.

## Materials and methods

### Study design and study population

The NAVIGATE study (MSD IDREC 947232) employed mixed-methods, comprising an online survey and semi-structured interviews, with UK adults who self-reported as sexual health service users. The study was grounded in a pragmatic paradigm, enabling the integration of quantitative and qualitative data to comprehensively explore both the prevalence and contextual drivers of vaccine attitudes. Attitudes, perceptions and acceptability of a 4CMenB vaccine repurposing for gonorrhoea prevention were examined. Eligible participants were aged ≥18 years, UK residents, with a history of using sexual health services. Disclosure of sexual behaviour or infection history was not a requirement for participation to minimise participant burden and protect privacy when engaging with already sensitive topics. Interview participants received a £25 voucher in line with National Institute for Health and Care Research guidance; survey participants were not reimbursed due to minimal burden. The research team was comprised of individuals with backgrounds in health sociology, sexual health and clinical vaccine research. Reflexivity was supported through regular team discussions and integration of public contributor perspectives during the design of study materials. To support transparency, various reporting tools were used; a Consensus-Based Checklist for Reporting of Survey Studies (CROSS) for the survey, Standards for Reporting Qualitative Research for the interviews, and Good Reporting of A Mixed-Methods Study (GRAMMS) for the mixed m-methods integration are included in online supplemental material 5.

### Patient and public involvement

Two adult public contributors with lived experience of UK sexual health services supported the development of the research aims and shaped the focus on vaccine accessibility, stigma and communication. They contributed to the design of the survey and interview guides, reviewing wording, tone and structure to ensure sensitivity and clarity. Public contributors also advised on recruitment materials and channels, helping to tailor posters and social media posts to relevant audiences. They reviewed the estimated time required for survey and interview participation and confirmed the burden was appropriate. Although not involved in data collection, they played an active role in ensuring the study was respectful and participant-centred.

### Sampling and sample size

For the quantitative component, we calculated the required sample size to estimate the proportion of health service users who would accept a gonorrhoea vaccination programme with adequate precision. Using the standard formula for population proportions:


$$n=\left[z^{2}\alpha /2\times p(1-p)\right]/E^{2}$$

Where n is the required sample size, z²α/2=1.96 for 95% confidence level, p=0.5 (conservative estimate for maximum variability when the true acceptance rate is unknown) and E=0.05 (5% margin of error).^29^ This calculation yielded a target sample size of 385 participants. To account for potential incomplete responses, we applied a 25% oversampling buffer, consistent with recommendations for online survey research.^30^

For the qualitative component, we employed a mixed sampling approach. Initially, we contacted interview participants sequentially from the list of survey respondents who expressed interest. However, recognising that early respondents were all of a similar demographic, we subsequently adopted purposive sampling to improve demographic variation, particularly seeking participants with diverse educational backgrounds and ethnicities where possible. We aimed for thematic saturation with an initial target of 12 interviews, based on empirical evidence suggesting that 9–17 interviews typically achieve saturation in studies with relatively homogeneous populations and focused research objectives,^31^ with Guest *et al* demonstrating that 92% of themes are typically identified within the first 12 interviews.^32^

### Data handling and storage

Survey data were collected using a bespoke REDCap (version 5.0.35) database. Survey links were distributed through displaying posters in various sexual health clinics and hospitals across the UK, as well as through paid and organic social media posts. Participants were first presented with a participant information sheet outlining the purpose of the research, their rights and data protection procedures. Participants were not permitted to proceed without first confirming their consent.

The survey instrument (online supplemental material 2) was structured into five key sections. First, demographic questions, then the validated Vaccination Attitudes Examination (VAX) scale,^33^ which assesses general attitudes toward vaccination across four domains: mistrust of vaccine benefit, worries about unforeseen future effects, concerns about commercial profiteering and preference for natural immunity. Subsequent sections focused on domain-specific content. The third section explored attitudes toward vaccination in the context of sexual health, the fourth section examined attitudes toward a potential gonorrhoea vaccination programme using the 4CMenB vaccine, and the final section focused on vaccine communication and education. Overall, the survey was estimated to take around 15 min to complete.

On completion, participants were invited to leave their email address if they were willing to be contacted for a follow-up interview. Selected participants were invited to participate in a one-to-one semi-structured interview lasting approximately 30–45 min. These were conducted on Microsoft Teams and sought to gain deeper insight into participants’ views, experiences and informational needs related to gonorrhoea vaccination and broader sexual health vaccination contexts. Interviews were conducted online, audio recorded with consent, transcribed verbatim, and anonymised via transcription, and followed a topic guide (online supplemental material 3). The topic guide was developed iteratively by the research team and through review with the public contributors. It was informed by the study objectives, existing literature on vaccine acceptability and the content of the online survey.

### Data analysis

Quantitative data were exported from REDCap and were analysed using IBM SPSS Statistics (V.30.0.0.0 (171)). Prior to analysis, the dataset was cleaned to remove incomplete responses and checked for inconsistencies. Descriptive statistics were used to summarise participant characteristics and item responses. Frequencies and percentages were calculated as appropriate. The free-text answers were printed out and key recurring ideas and themes were highlighted.

Qualitative data from the semi-structured interviews were analysed thematically using NVivo (V.15) following the six-phase approach outlined by Braun and Clarke.^34^ Transcripts were first read in full to ensure familiarity, followed by open coding of the text line-by-line. Initial codes were inductively generated and then grouped into broader categories through a process of constant comparison. A coding framework was iteratively developed and refined through regular discussions among members of the research team.

Data integration was undertaken through triangulation of the survey and interview findings. Quantitative results were used to inform and contextualise qualitative insights, and vice versa, enabling a more comprehensive understanding of participants’ attitudes and perceptions. This integration enabled interpretation of survey findings through the lens of lived experience, revealing how structural and social barriers shaped otherwise high levels of stated vaccine acceptability.

## Results and discussion

### Participant characteristics

Between 6 April and 1 May 2025, a total of 500 individuals initiated the online survey. Following the exclusion of incomplete responses, 395 fully completed surveys (10 more than the minimum required of 385) were retained for analysis. The demographic characteristics of these participants are presented in table 1. The sample was predominantly male (92.7%), aged 25–44 years (80.0%) and White British/Irish (72.7%). Most participants were employed full-time (77.7%) and had no children (95.7%).

**Table 1:** Sociodemographic of survey participants

Characteristic	N=395	Percentage
Age		
25–34 years	164	41.50
35–44 years	152	38.50
45–54 years	54	13.70
55–64 years	13	3.30
18–24 years	8	2.00
65 years and over	4	1.00
Sex		
Male	366	92.70
Female	16	4.10
Non-binary or other	12	3.00
Prefer not to say	1	0.30
Ethnicity		
White (British or Irish)	287	72.70
White (other European)	66	16.70
Asian or Asian British (eg, Indian, Pakistani, Bangladeshi)	14	3.50
Mixed (eg, White and Black Caribbean, White and Black African, white and Asian)	11	2.80
Other	9	2.30
Black or Black British (eg, Caribbean, African)	4	1.00
Chinese	4	1.00
Religion		
None	287	72.70
Christian (eg, Church of England, Catholic, Protestant)	57	14.40
Prefer not to say	23	5.80
Buddhist	8	2.00
Muslim	7	1.80
Other	6	1.50
Jewish	5	1.30
Hindu	1	0.30
Sikh	1	0.30
Employment status		
Employed full-time	307	77.70
Self-employed	35	8.90
Employed part-time	20	5.10
Student	16	4.10
Unemployed	13	3.30
Prefer not to say	4	1.00
Number of children		
None	378	95.70
One child	9	2.30
Two children	5	1.30
Three or more children	3	0.80
Highest level of education		
Master’s degree/post-graduate diploma/level 7	184	46.60
Undergraduate degree/HND/level 6	107	27.10
PhD	47	11.90
A-levels/level 3 BTEC/NVQ	31	7.80
Foundation degree/diploma/level 5	13	3.30
GSCE/O-level/CSE	10	2.50
Other	2	0.50
No formal qualifications	1	0.30

BTEC, Business and Technology Education Council; CSE, Certificate of Secondary Education; GCSE, General Certificate of Secondary Education; HND, Higher National Diploma; NVQ, National Vocational Qualification.

A total of 15 individuals took part in a semi-structured interview; the full demographic characteristics of these participants are presented in table 2. The sample largely identified as male (80%), was aged 25–34 years (60%) and was White British/Irish (53.3%). 66.7% of participants had no religion and 73.3% were employed full-time. All of the participants had no children, and most had either a postgraduate master’s degree (40%) or an undergraduate degree (33.3%) (or equivalent).

**Table 2:** Demographic of interview participants

Characteristic	N=15	Percentage
Age		
25–34 years	9	60
35–44 years	4	26.70
45–54 years	2	13.30
Sex		
Male	12	80
Female	2	13.30
Non-binary or other	1	6.70
Ethnicity		
White (British or Irish)	8	53.30
White (other European)	3	20
Mixed (eg, White and Black Caribbean, White and Black African, white and Asian)	2	13.30
Asian or Asian British (eg, Indian, Pakistani, Bangladeshi)	1	6.70
Black or Black British (eg, Caribbean, African)	1	6.70
Religion		
None	10	66.70
Christian (eg, Church of England, Catholic, Protestant)	2	13.30
Prefer not to say	2	13.30
Muslim	1	6.70
Employment status		
Employed full-time	11	73.30
Self-employed	2	13.30
Student	1	6.70
Unemployed	1	6.70
Number of children		
None	15	100
Highest level of education		
Master’s degree/post-graduate diploma/level 7	6	40
Undergraduate degree/HND / level 6	5	33.30
A-levels/level 3 BTEC/NVQ	3	20
GSCE/O-level/CSE	1	6.70

BTEC, Business and Technology Education Council; CSE, Certificate of Secondary Education; GCSE, General Certificate of Secondary Education; HND, Higher National Diploma; NVQ, National Vocational Qualification.

### Attitudes to vaccines (VAX scale)

#### Trust in vaccines

Participants showed high confidence in the effectiveness and safety of vaccines. A majority strongly agreed that they feel safe after being vaccinated (72.3%), with the remaining 21.8% agreeing. Nearly all (92.5%) strongly agreed or agreed that they can rely on vaccines to stop serious infectious diseases, and 92.9% strongly agreed or agreed that they feel protected after vaccination. These findings suggest widespread trust in the core function of vaccines as preventive health tools, with very few participants expressing doubt or uncertainty about their protective value.

#### Worries about unforeseen future effects

Concerns about long-term or undiscovered vaccine side effects were relatively low among participants. Only a small proportion strongly agreed that vaccines may have yet-to-be-discovered problems (8.4% strongly agree, 12.9% agree, 30.1% somewhat agree), could cause unforeseen problems in children (1% strongly agree, 1.3% agree, 16.7% somewhat agree), or might have unknown effects in the future (1.3% strongly agree). In contrast, a noteworthy number strongly disagreed with these concerns, particularly regarding unknown future effects (44.0%). This indicates that, while a minority hold cautious views, most participants were not influenced by fear of hidden or long-term harms.

#### Concerns about commercial motives

Scepticism towards the motives of pharmaceutical companies or health authorities was limited. Just over half of participants (53.1%) strongly disagreed that vaccines mainly benefit companies rather than people, and 73.7% strongly disagreed that vaccination is promoted for financial gain rather than health. The statement that ‘vaccination programmes are a big con’ received even less endorsement, with an overwhelming 86.6% strongly disagreeing. These results reflect a high level of institutional trust and low endorsement of profit-driven or even conspiratorial narratives.

#### Preference for natural immunity

**Figure 1:**
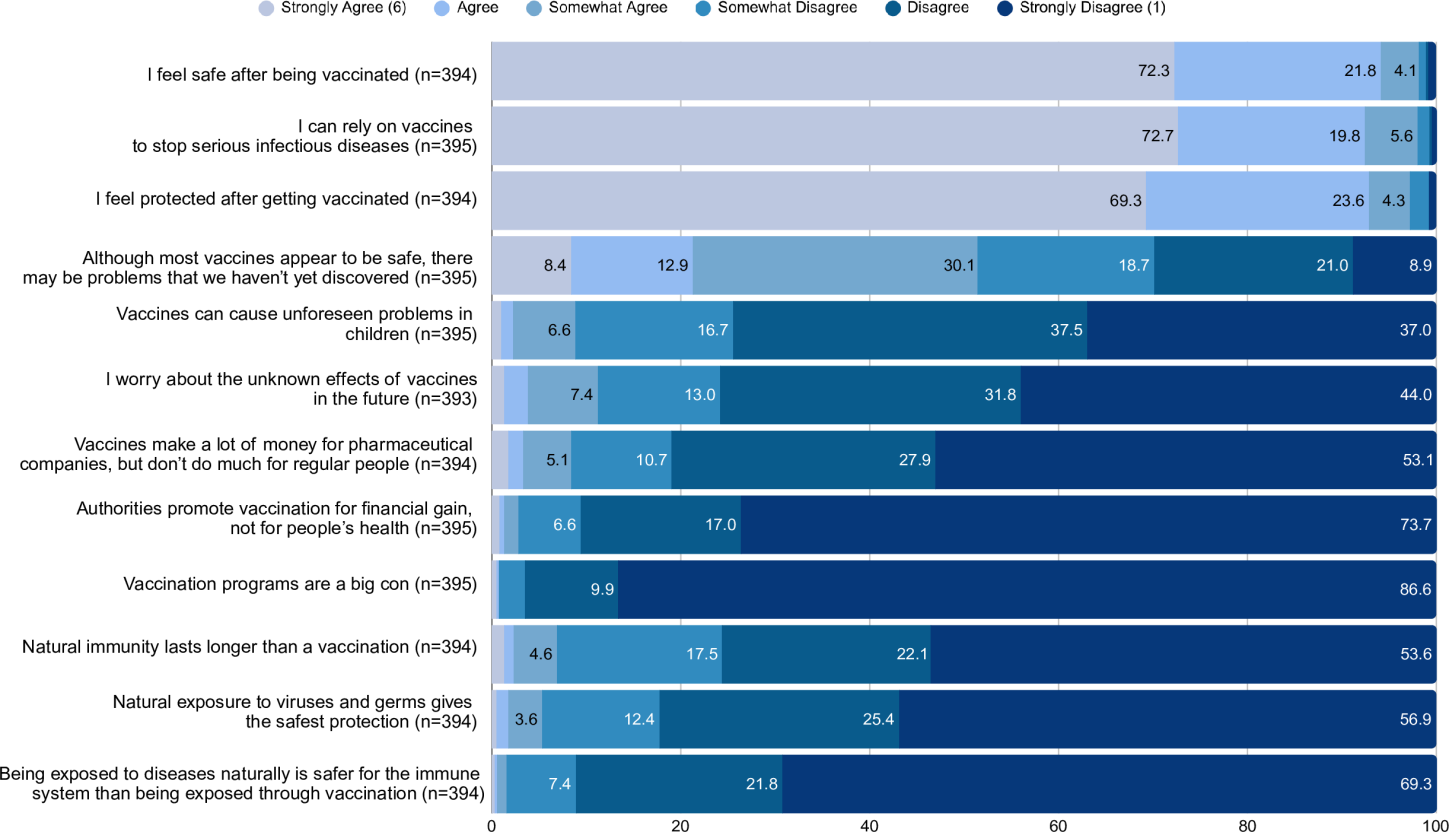
Stacked bar chart showing participant responses to the Vaccination Attitudes Examination (VAX) scale. The bars represent the distribution of responses across each VAX subscale item, illustrating levels of agreement or disagreement. Colours correspond to Likert-scale categories, from strong disagreement to strong agreement. Percentages are rounded to one decimal place and may not sum to exactly 100% due to rounding.

Participants generally rejected the idea that natural exposure is superior to vaccination. The majority strongly disagreed that natural immunity lasts longer than vaccine-induced immunity (54.6%) or that natural exposure gives the safest protection (56.9%). Most participants (61.9%) disagreed that natural exposure is safer for the immune system. Overall, the VAX scale responses demonstrate strong vaccine confidence and low levels of mistrust or hesitancy, reinforcing that any concerns about a gonorrhoea vaccine are likely to be programme-specific rather than ideologically rooted (figure 1).

### Gonorrhoea vaccination programme acceptability

#### Overall support and programme features

Participants had the option to read a fact sheet about using meningitis B vaccines as gonorrhoea vaccines before completing the survey (online supplemental material 1). Support for gonorrhoea vaccination was nearly universal, with 92.2% strongly agreeing to the introduction of the programme. In the free-text answers, one participant stated: “I would walk through fire to get this vaccine, this would be a huge improvement to the sexual health of LGBT people” (Male, 35–44). Over half of the participants strongly endorsed the programme for targeting high-risk populations (51.2%), with a slightly higher proportion (63.7%) preferring an adolescent vaccine programme.

#### Efficacy concerns and reservations

**Figure 2:**
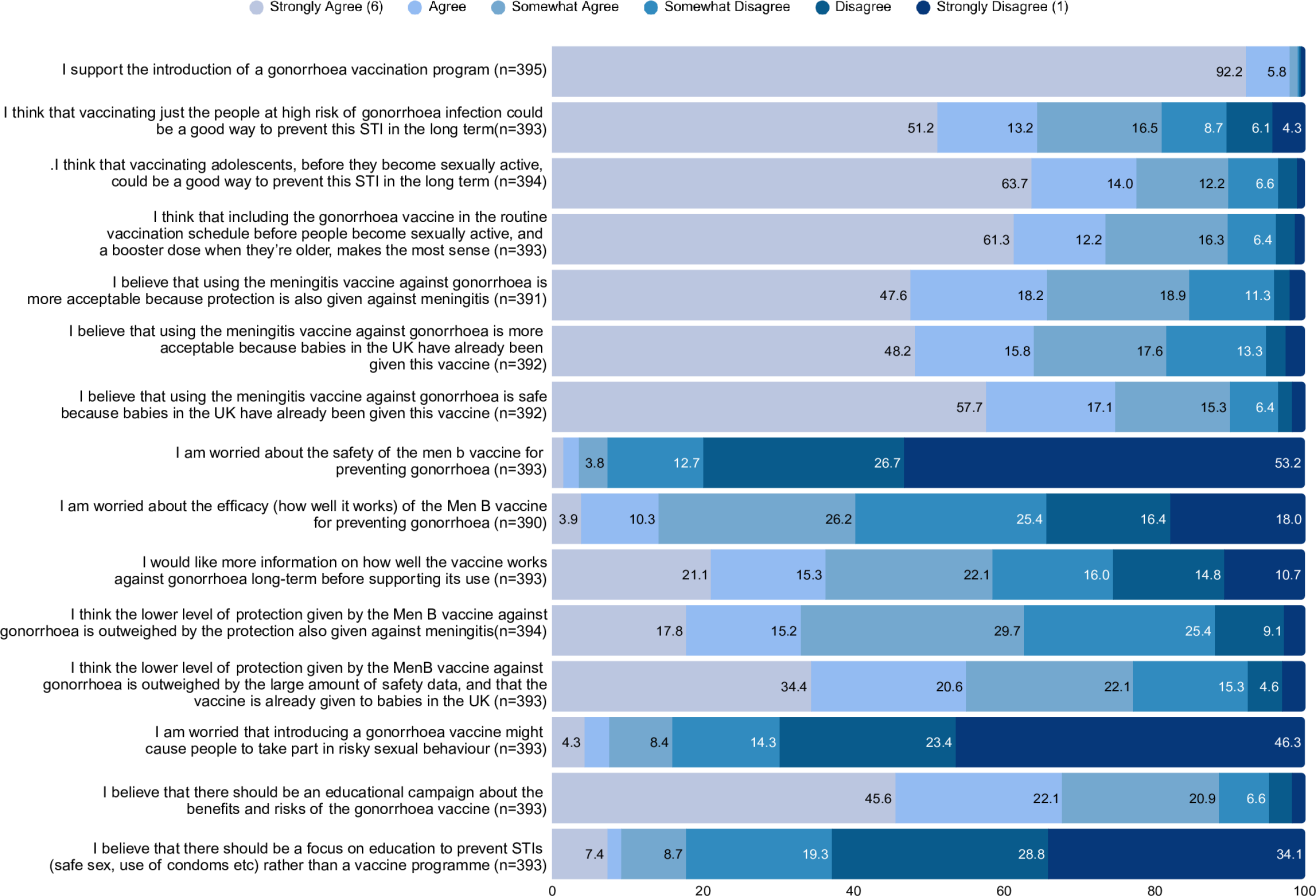
Stacked bar chart illustrating participant attitudes toward a potential gonorrhoea vaccine programme. Responses are grouped by level of agreement with key statements related to vaccine acceptability, delivery preferences and perceived need. Colours represent Likert-scale categories from strong disagreement to strong agreement. Percentages are rounded to one decimal place and may not sum to exactly 100% due to rounding. STIs, sexually transmitted infections.

Despite overall support, efficacy concerns emerged as the primary reservation. Just 34.4% of respondents responded strongly disagree, or disagree, in response to the statement, ‘I am worried about the efficacy of the Men B vaccine for preventing gonorrhoea’. The remaining 3.9% strongly agreed, 10.3% agreed, 26.2% somewhat agreed and 25.4% somewhat disagreed. However, to some extent, this was mitigated by the dual protection also offered against meningitis (17.8% strongly agree, 15.2% agree, 29.7% somewhat agree). Just over half (57.7%) of participants strongly agreed that the 4CMenB vaccine is safe, as it is already used in babies. The mixed responses highlight an important tension: even among a population broadly supportive of vaccination, uncertainty around vaccine purpose, performance and public messaging contributed to conditional forms of acceptance, and 45.6% of participants strongly agreed that an educational campaign that explains the benefits and risks of gonorrhoea vaccination is necessary. Full item-level responses are presented in figure 2.

### Attitudes and perceptions towards a gonorrhoea vaccine programme in the semi-structured interviews

#### AMR and sexual behaviour as programme justifications

Throughout the semi-structured interviews, participants broadly perceived the introduction of a vaccine against gonorrhoea as a necessary and timely public health intervention, primarily due to escalating concerns about AMR. The threat of gonorrhoea becoming untreatable was a key motivating factor in vaccine acceptance, with respondents framing AMR as an urgent risk necessitating a preventative solution: “There’s a degree of antibiotic resistance to certain infections … whatever we can do to cut down the spread of them is probably the most sensible thing” (Male, 45–54). This perspective clearly demonstrates participants’ pragmatic recognition of a need to mitigate the escalating public health threat posed by AMR. The idea that gonorrhoea could become untreatable was described as both credible and frightening: “The fact that this is becoming incurable would scare people … they need to be scared, and they should be scared realistically” (Female, 25–34). A minority advocated for deliberately provocative public health messaging to grab attention and shift perceptions: “Whatever we give you, it’s not going to work. Your [genitals] will melt … I think that could be a really good campaign hook” (Male, 35–44 years). However, this shock-based approach was tempered by concerns about misinformation. Participants were warned against framing that might exaggerate the vaccine’s efficacy: “You’d need to avoid a confusing message where people believe … they’re some kind of Superhuman who’s never going to catch either” (Male, 35–44). This pragmatism extended beyond the health messaging into participants’ broader understanding of population health, with several participants explicitly linking AMR to the need for pre-emptive, system-wide action*: “*If it becomes completely antibiotic resistant and then spreads like wildfire through the community … we’re going to be in a really scary, very dangerous place” (Male, 35–44).

Such views were closely tied to an acknowledgement that current behavioural prevention methods, especially condoms and oral prophylaxis such as HIV pre-exposure prophylaxis (PrEP), are inherently limited by user adherence and situational constraints: “[Condoms] are effective if used. They’re very user dependent. … It’s all event based” (Male, 25–34), and “You forgot [PrEP] on holiday … your protection goes down. So if there is anything that could be administered once and worked for [a] prolonged period … that would be really helpful” (Male, 35–44). Participants explicitly highlighted the idea of risk compensation associated with biomedical interventions such as HIV PrEP. While HIV PrEP was recognised as highly effective in preventing HIV transmission, several participants observed that its availability had reduced condom usage, inadvertently increasing susceptibility to other sexually transmitted infections: “Clearly one of the things about the availability of [HIV] PrEP now is that it clearly helps to alleviate one problem, but it makes the problem of sexually transmitted infection with other infections more likely now” (Male, 35–44). Overall, participants viewed vaccination as a complementary strategy capable of mitigating the real-world limitations of individual adherence.

#### Acceptance rooted in trust, transparency, confidence and safety

The acceptability of a gonorrhoea vaccine was underpinned by a strong degree of institutional trust. Participants consistently expressed confidence in UK regulatory processes to ensure the safety, efficacy and ethical deployment of vaccines*:* “I have a lot of confidence in the UK, in regulating them to make them safe … I don’t know the data, but I know there will be lots of data behind [it] because they wouldn’t be approved as [a] vaccine in the UK otherwise” (Male, 25–34). This participant’s quote underscores the broader theme of institutional trust, highlighting how confidence in regulatory authorities strongly supports vaccine acceptance among the target population. Further confidence was derived from the MenB vaccine’s prior use in infants, a population perceived as particularly vulnerable. This history of safe administration enhanced participants’ belief in its safety for adult populations: “Because the vaccine has already been in circulation for a different disease, it’s already proved to be non-harmful” (Non-binary/other, 25–34). The potential for dual protection, against both MenB and gonorrhoea, was received positively when framed as an unexpected advantage: “It’s like … opening your Cornetto ice cream box where they say … it’s five and you find the sixth one” (Male, 35–44). But participants did express concern about overwhelming people with excessive information in a single communication: “If you push too much information in one go … the message … disappears” (Male, 35–44). Messaging around vaccine efficacy also required careful calibration.

While participants understood that partial efficacy was still meaningful*:* “40% is better than zero… the question is, is it in preventing [gonorrhoea] completely or preventing the severity of the disease? Because even if it’s, you know, it’s 33% [effective] at preventing [gonorrhoea] completely. That’s not bad at all” (Male, 35–44). But this perception was not unanimous; some indicated that a low protection rate would necessitate greater personal deliberation: “If it’s only 40%, I think I would definitely take more time to consider” (Female, 25–34). Participants favoured messages that were transparent and grounded in evidence, avoiding vague or euphemistic language often associated with public health campaigns. They saw clarity as a way to empower rather than frighten: “It’s not about panicking people, but people need to actually understand what this infection can do because most people think it’s just an antibiotic and done” (Male, 35–44). There was also recognition that effective communication should challenge the normalisation of risk, particularly in relation to practices such as condom-less oral sex, which many participants noted were common yet rarely discussed in public health materials: “One of the risk factors for contracting gonorrhoea … will be people engaging in unprotected oral sex and that’s probably the majority of people who practise oral sex anyway” (Male, 35–44).

#### Stigma and cost as structural barriers to access

Stigma emerged as a complex barrier to vaccine delivery, shaping where the participants felt safe accessing it. For many, discomfort stemmed from the broader sociocultural narratives that continue to frame certain sexual behaviours as deviant from the norm. Participants expressed fears of being judged by healthcare providers, particularly in settings where sexual health was not routinely discussed, rooted in past experiences or historical memory, particularly among gay men who had lived through the HIV/AIDS epidemic. One participant’s reflection powerfully captured this intersection of stigma, history and healthcare access:

It’s not just the judgment. As a gay man growing up in the 90s at the height of the AIDS pandemic, there is stigma and there is still a lot of negative advertising around gay sex and promiscuity. I don’t want people in places like pharmacies or GP providing less appropriate service because of what they think of me. (Male, 35–44)

This illustrates how stigma continues to influence not only perceptions of care but also the actual behaviour of service users. The lingering effects of AIDS-era rhetoric have, for some, entrenched a sense of vulnerability and conditional belonging within mainstream healthcare services. This led to strong preferences for Sexual Health or Genitourinary Medicine (GUM) clinics delivering the gonorrhoea vaccine programme, which were considered less judgemental than general practices or high-street pharmacies: “Any GUM clinic would work. I definitely don’t want to do it at my local pharmacy” (Male, 35–44). Some participants referred to prior outreach initiatives as evidence that non-clinical settings could support engagement with the vaccine programme*:* “Perhaps in bars or clubs, potentially in gay saunas in particular … sexual health clinics do pop up in those places … that’s probably well worth considering” (Male, 35–44). Alongside this, a clear preference emerged for peer-to-peer communication, particularly from individuals with shared identities or lived experiences. This was grounded in trust, relatability and perceived authenticity: “I would definitely trust fellow gays … definitely gay influencers … people on other platforms like TikTok or Instagram and whatnot who are very openly gay and who talk about, trials and tribulations of being gay” (Male, 35–44). The use of prominent queer public figures, especially those with a track record of advocacy, was also described as powerful in increasing visibility and acceptance of the vaccine programme*:* “I’d love to see those, you know, Sir Ian McKellen, on one of those posters … going grassroots could really have a great effect” (Male, 35–44).

However, some participants explicitly stated that local pharmacies would be a more convenient option: “I think pharmacy is convenient and they’re, you know, they’re everywhere” (Male, 35–44), with others also emphasising how important ease of getting the vaccine in their decision to take it: “I just want to book in with a nurse, get the jab, out I go. … I don’t want to have to travel half an hour … because I will likely just take the risk” (Female, 25–34). Participants expressed discomfort with having to justify their request for a sexual health vaccine: “I don’t want to have to sit in the doctor, talk about why I’m doing this. Obviously, if I want to be protective against gonorrhoea because I wanna have sex, that’s it” (Female, 25–34). In this way, stigma functioned as both a potential psychological and structural barrier to uptake. Another perceived structural barrier to vaccination against gonorrhoea was cost; while some participants stated they could personally afford to pay: “Even if there were just a nominal cost to the vaccine, I think I would gladly bear it” (Male, 35–44), others emphasised how even modest costs would disproportionately exclude vulnerable populations: “I would [pay], but … if there were a cost associated with it, a lot of people [would] forgo it … someone on 21–24 k a year, where every penny counts” (Male, 35–44). Some participants explicitly invoked the current economic realities affecting them: “People already don’t have enough money for things like food. … I’m not gonna drop £25 on a vaccine” (Female, 25–34). These perspectives amalgamated into a strongly articulated belief that preventive sexual health services should be considered a basic entitlement: “Health in general is a human right … Nobody should be rejected … on the basis of their financial positioning” (Female, 25–34), highlighting the public’s perceived value of a vaccine capable of reducing gonorrhoea cases. A summary table of qualitative findings is provided in online supplemental material 4.

## Discussion

This mixed-methods study found broad support among sexual health service users in the UK for a gonorrhoea vaccination programme using a 4CMenB vaccine. Support was influenced by concerns regarding AMR, trust in UK healthcare bodies, equitable access, previous stigmatisation of marginalised groups in healthcare access, and targeted communication and outreach strategies.

The participants in this study predominantly identified as male and were aged under 55 years. It is plausible that the predominance of male respondents reflects a perception of gonorrhoea as largely a male issue, or at least more salient for GBMSM communities. Women may not perceive themselves at risk for STIs, even when prevalence is non-trivial, particularly because infection may be asymptomatic. For example, a UK national survey found that 73% of women rated themselves as ‘not at all at risk’ for STIs, compared with lower rates among men (64%), even though women may experience serious complications as a result of gonorrhoea, including infertility.^35^

### Vaccine attitudes and programme acceptance

VAX scale findings indicated that, in this group of participants, vaccine hesitancy stems from context-specific factors rather than ideological concerns. Participants distinguished between general vaccine confidence and programme-specific reservations, suggesting concerns are addressable through transparent communication rather than requiring fundamental opposition strategies. AMR emerged as the primary driver of acceptance, with participants framing vaccination as a suitable biomedical intervention. This aligns with international public health AMR discourse.^36^ Participants recognised behavioural interventions (condoms, PrEP) as adherence-limited, reinforcing vaccination as complementary prevention. As similarly observed during the development of COVID-19 vaccines,^37^ acceptance in the gonorrhoea vaccine programme was underpinned by institutional trust in UK regulatory processes, bolstered by 4CMenB’s established safety record.^38^ These findings are consistent with existing literature demonstrating that trust in the quality and safety of vaccines, and in the institutions responsible for their delivery, is critical to willingness to be vaccinated.^17^

However, efficacy concerns highlight the need for clear, realistic messaging about vaccine benefits to prevent misconceptions. Perceptions of universal accessibility emerged as essential, highlighting the high value participants placed on this programme, with some identifying potential cost as a barrier to receiving the vaccine, and therefore worsening inequalities. This notion of universal accessibility reflects the broader NHS Constitution for England,^39^ which emphasises equitable provision imperatives supported by health equity research. This is also consistent with wider health equity reports demonstrating that financial and structural barriers can undermine the reach and impact of preventive health interventions.^40^

### Programme delivery and communication

Stigma represented potential significant psychological and structural barriers, with various GBMSM (as self-identified during the semi-structured interview) participants expressing preferences for specialised sexual health services over general healthcare settings, as they were perceived as less stigmatising and more affirming than general healthcare settings. Participants emphasised that destigmatising efforts would be most effective when combined with local, grassroots strategies, such as drop-in clinics, and events held in partnership with LGBTQ+ venues. These community-based approaches are seen as crucial in making healthcare services more accessible and welcoming.^41^

In addition to destigmatising vaccine delivery, strong endorsement emerged for peer-led dissemination of information through community leaders, influencers and grassroots organisations, considered central rather than supplementary to institutional efforts.^42^ The emphasis on peer-to-peer communication, particularly within LGBTQ+ communities, suggests a viable avenue for promoting vaccine acceptance. Peer-led activism and community groups were regarded as highly persuasive, particularly for populations already marginalised within health systems. As demonstrated by Biesty *et al*,^24^ engagement through trusted community groups and visible peers can enhance credibility and acceptability of public health interventions among groups with prior experiences of marginalisation. Well-known figures from within the LGBTQ+ community were also seen as uniquely positioned to bridge the gap between clinical settings and community realities, and as suggested in previous research, queer celebrities are powerful agents for raising awareness of LGBTQ+ issues.^43^ Overall, community mobilisation and peer influence were considered central to effective dissemination alongside institutional efforts, particularly for reaching those who might otherwise distrust formal systems, avoid clinical encounters due to stigma or simply not engage with conventional public health messaging.

### Policy recommendations

To ensure the success of a gonorrhoea vaccination programme, these findings suggest some recommendations which include targeting actions across delivery, communication and outreach. The vaccine should be offered free of charge and delivered through trusted settings such as sexual health clinics and mobile pop-ups in LGBTQ+ venues, which were perceived as less stigmatising. Low-barrier access models should be integrated into routine sexual health appointments. Communication should be transparent about the vaccine’s partial efficacy, framing it as a meaningful layer of protection, as part of a wider package of preventative measures. Peer-led campaigns featuring LGBTQ+ influencers could be successful in building trust, normalising uptake and extending reach to those not engaged with traditional health messaging in this community. Finally, real-time monitoring of uptake across demographic groups would be critical to ensure equity and inform responsive policy adjustments.

### Limitations

Several limitations should be noted in interpreting these findings. First, a limitation of the mixed-methods design is that while the qualitative data added valuable depth to interpretation, it was collected from a smaller and more demographically homogeneous subset of survey participants, which may limit representativeness. Further, the majority of participants in this study identified as male, reflecting the targeted focus of the gonorrhoea vaccination programme towards GBMSM. While our predominantly male sample reflected the epidemiological concentration of gonorrhoea among GBMSM in the UK, including more women may have yielded different perceptions of vaccine acceptability, given that there are broader implications of infection which extend to other demographics. Modelling studies have also shown that adolescent vaccination programmes could provide substantial population-level protection,^44^ and so future research should explore the acceptability of adolescent gonorrhoea vaccination or population-wide approaches.

Second, sexual orientation was not explicitly collected from participants; however, the inclusion criterion of prior experience with sexual health services aligns well with the programme’s broad target demographic—individuals at elevated risk of bacterial STIs who attend sexual health clinics. Additionally, the online recruitment strategy may have introduced self-selection bias, potentially attracting individuals with stronger or more polarised views on vaccination, thus affecting the representativeness of the findings.

Finally, although the quantitative sample was statistically powered to ensure accuracy and reliability, it was not stratified by specific demographics. Therefore, findings should be interpreted with caution when generalising beyond the sampled population. Future research should explore vaccine perceptions, knowledge and acceptability among a broader cross-section of the population, including women, adolescents and groups that are traditionally underserved by sexual health services, to inform more inclusive and effective public health strategies. These include those from lower socioeconomic communities, minority ethnic groups, and refugees and asylum seekers.^45 46^

## Conclusion

The findings of this study demonstrate high overall acceptability of the 4CMenB vaccine among the study participants, particularly when delivery is trusted, stigma-free and supported by transparent communication and peer-led outreach. As the world’s first gonorrhoea vaccination programme, the UK will provide real-world evidence for international policy development.

This study highlights the importance of addressing intersecting behavioural, structural and social factors that influence vaccine acceptability and access. To support equitable and effective delivery, we recommend specific policy actions, including ensuring free and universal access, prioritising delivery through trusted low-stigma settings, implementing peer-led outreach and clearly communicating the vaccine programme’s potential benefits and limitations. Monitoring of uptake across diverse populations will be critical to guide responsive adjustments and prevent disparities. Monitoring is needed to evaluate the impact of population-wide vaccination approaches and refine targeting strategies. Lessons from this pioneering programme will shape future responses to the growing challenge of antimicrobial-resistant gonorrhoea and the broader role of vaccination in STI control.

## Supplementary material

10.1136/bmjph-2025-003819 online supplemental file 1

10.1136/bmjph-2025-003819 online supplemental file 2

10.1136/bmjph-2025-003819 online supplemental file 3

10.1136/bmjph-2025-003819 online supplemental file 4

10.1136/bmjph-2025-003819 online supplemental file 5

## Data Availability

No data are available.
