# Supplementary material for: “I would walk through fire to get this vaccine”: a mixed-methods study examining attitudes and perceptions of a gonorrhoea vaccine programme among UK sexual health service users
Source: BMJ Public Health. 2026 Mar 27;4(1):e003819. doi: 10.1136/bmjph-2025-003819 (PMC13034242; doi:10.1136/bmjph-2025-003819)
Supplement: online supplemental file 1 [file bmjph-4-1-s001.pdf]

Men B as a Gonorrhoea Vaccine Fact Sheet

The 4CMenB vaccine is used to prevent meningitis B – a potentially life-threatening bacterial infection that can cause swelling of the brain and spinal cord. The licensed version of this vaccine, Bexsero® is given to babies in the UK. The '4C' means the vaccine contains four components, or four parts, from the bacteria that cause Meningitis B.

There are some similarities between the meningitis B bacteria and the bacteria that cause gonorrhoea. Vaccines that contain these similar parts, such as the 4CMenB vaccine, have been shown to prevent both meningitis and gonorrhoea.

The 4CMenB vaccine is referred to as the Men B vaccine in the rest of this fact sheet.

In November 2023, the Joint Scientific Committee on Vaccination and Immunisation (JCVI) recommended the use of Men B vaccine to prevent gonorrhoea in people who are more likely to get an STI (especially gay, bisexual, and other men who have sex with men).

As of October 2024, none of the JCVI's recommendations for a Men B vaccine gonorrhoea prevention programme have been implemented in the UK.

Mathematical modelling studies that aim to predict the potential impact of vaccination programmes have shown that vaccinating teenagers, alongside a booster programme for young adults, could also be effective at preventing cases of gonorrhoea.

Key Facts

- Research shows that the Men B vaccine can prevent gonorrhoea.
- The vaccine was originally designed to protect people against meningococcal B bacteria – the bacteria that causes some type of meningitis.
- Evidence suggests that the Men B vaccine has between 32.7 to 42% effectiveness against gonorrhoea.
- The vaccine could be particularly useful for people who are at the highest risk of getting gonorrhoea.
- The vaccine could also help save the NHS time and money by reducing the number of cases of gonorrhoea that need to be treated.
- The vaccine, if effective at preventing gonorrhoea in the UK, could slow the increase in antimicrobial-resistant cases of gonorrhoea – sometimes also called 'super gonorrhoea'.
- Vaccination for gonorrhoea prevention is not yet available on the NHS.
