# Supplementary material for: “I would walk through fire to get this vaccine”: a mixed-methods study examining attitudes and perceptions of a gonorrhoea vaccine programme among UK sexual health service users
Source: BMJ Public Health. 2026 Mar 27;4(1):e003819. doi: 10.1136/bmjph-2025-003819 (PMC13034242; doi:10.1136/bmjph-2025-003819)
Supplement: online supplemental file 2 [file bmjph-4-1-s002.pdf]

NAVIGATE: Navigating Attitudes to Vaccination Implementation for Gonorrhoea in the UK: Acceptability, Trust and Engagement

Sexual health service user survey

Introduction and Consent Confirmation

Welcome to the survey!

Thank you for participating in this survey about your thoughts about vaccines for gonorrhoea. This survey is designed to gather your opinions on vaccinations, particularly in the context of sexual health vaccines. Your responses will help guide decisions on future vaccine programs.

Your answers are private and will only be used for research purposes. No personally identifiable information will be linked to your responses.

Taking part in this survey is entirely voluntary. You can skip any question you do not wish to answer or exit the survey at any time.

Consent: By clicking "Next" and proceeding with this survey, you confirm that you have read and understood the participant information sheet provided and you consent to participate in this survey.

Demographic Information

1. **Do you identify as...?**
   - Female
   - Male
   - Non-binary or other
   - Prefer not to say

2. **Age Group:**
   - Under 18 years
   - 18-24 years
   - 25-34 years
   - 35-44 years
   - 45-54 years
   - 55-64 years
   - 65 years and over.

3. **Ethnic Group:**
   - Asian or Asian British (e.g., Indian, Pakistani, Bangladeshi)
   - Black or Black British (e.g., Caribbean, African)
   - Chinese
   - Mixed (e.g., White and Black Caribbean, White and Black African, White and Asian)
   - White (British or Irish)

- White (Other European)
- Other
- Prefer not to say

3a. If you selected "Other," please specify:

4. **Religion:**
   - Buddhist
   - Christian (e.g., Church of England, Catholic, Protestant)
   - Hindu
   - Jewish
   - Muslim
   - Sikh
   - None
   - Other
   - Prefer not to say

   4a. If you selected "Other," please specify:

5. **First Half of Your Postcode:**

6. **Highest Level of Education:**
   - No formal qualifications
   - GSCE / O-Level / CSE
   - A-Levels / Level 3 BTEC / NVQ
   - Foundation Degree / Diploma / Level 5
   - Undergraduate Degree / HND / Level 6
   - Masters Degree / Post-graduate Diploma / Level 7
   - PhD
   - Other

7. **Employment Status**
   - Employed Full-Time
   - Employed Part-Time
   - Self-Employed
   - Unemployed
   - Student
   - Prefer not to say

8. **Household Income:**
   - Less than £15,000
   - £15,000 - £24,999
   - £25,000 - £34,999
   - £35,000 - £44,999
   - £45,000 - £54,999
   - £55,000 - £64,999
   - £65,000 or more
   - Prefer not to say

9. **Number and Ages of Children:**
   - None
   - 1 child (age)

- 2 children (ages)
- 3 or more children (ages)
- Prefer not to say

Section 1: General Attitudes Toward Vaccination (VAX – Vaccine Attitudes Examination)

These questions are designed to help us better understand people's beliefs about vaccinations. Please mark the choices that most accurately reflect your feelings or beliefs. There are no right or wrong responses.

1. **I feel safe after being vaccinated.**
   - Strongly Disagree (1)
   - 2
   - 3
   - 4
   - 5
   - Strongly Agree (6)

2. **I can rely on vaccines to stop serious infectious diseases.**
   - Strongly Disagree (1)
   - 2
   - 3
   - 4
   - 5
   - Strongly Agree (6)

3. **I feel protected after getting vaccinated.**
   - Strongly Disagree (1)
   - 2
   - 3
   - 4
   - 5
   - Strongly Agree (6)

4. **Although most vaccines appear to be safe, there may be problems that we haven't yet discovered.**
   - Strongly Disagree (1)
   - 2
   - 3
   - 4
   - 5
   - Strongly Agree (6)

5. **Vaccines can cause unforeseen problems in children.**
   - Strongly Disagree (1)
   - 2
   - 3
   - 4
   - 5

- Strongly Agree (6)

6. **I worry about the unknown effects of vaccines in the future.**
    - Strongly Disagree (1)
    - 2
    - 3
    - 4
    - 5
    - Strongly Agree (6)

7. **Vaccines make a lot of money for pharmaceutical companies, but don't do much for regular people.**
    - Strongly Disagree (1)
    - 2
    - 3
    - 4
    - 5
    - Strongly Agree (6)

8. **Authorities promote vaccination for financial gain, not for people's health.**
    - Strongly Disagree (1)
    - 2
    - 3
    - 4
    - 5
    - Strongly Agree (6)

9. **Vaccination programs are a big con.**
    - Strongly Disagree (1)
    - 2
    - 3
    - 4
    - 5
    - Strongly Agree (6)

10. **Natural immunity lasts longer than a vaccination.**
    - Strongly Disagree (1)
    - 2
    - 3
    - 4
    - 5
    - Strongly Agree (6)

11. **Natural exposure to viruses and germs gives the safest protection.**
    - Strongly Disagree (1)
    - 2
    - 3
    - 4
    - 5

- Strongly Agree (6)

12. **Being exposed to diseases naturally is safer for the immune system than being exposed through vaccination.**
    - Strongly Disagree (1)
    - 2
    - 3
    - 4
    - 5
    - Strongly Agree (6)

Section 2: Attitudes Toward Sexual Health Vaccination

These questions are designed to help us better understand your beliefs about sexually transmitted infections and vaccines. Please mark the choices that most accurately reflect your feelings or beliefs. There are no right or wrong responses.

Attitudes Toward Sexually Transmitted Infections (STIs)

1. **I believe sexually transmitted infections (STIs) are a serious public health issue.**
    - Strongly Disagree (1)
    - 2
    - 3
    - 4
    - 5
    - Strongly Agree (6)

2. **STIs can have severe long-term health consequences.**
    - Strongly Disagree (1)
    - 2
    - 3
    - 4
    - 5
    - Strongly Agree (6)

3. **The risk of contracting an STI is high among sexually active individuals.**
    - Strongly Disagree (1)
    - 2
    - 3
    - 4
    - 5
    - Strongly Agree (6)

4. **The risk of contracting an STI is high among individuals who have sexual contact with multiple people.**
    - Strongly Disagree (1)
    - 2
    - 3

- 4
- 5
- Strongly Agree (6)

5. **I am concerned about the spread of STIs in my community.**
    - Strongly Disagree (1)
    - 2
    - 3
    - 4
    - 5
    - Strongly Agree (6)

6. **STIs are preventable through proper education and preventive measures.**
    - Strongly Disagree (1)
    - 2
    - 3
    - 4
    - 5
    - Strongly Agree (6)

Attitudes Toward Vaccines for Sexually Transmitted Infections (STIs)

6. **Vaccines are an effective way to prevent sexually transmitted infections (STIs).**
    - Strongly Disagree (1)
    - 2
    - 3
    - 4
    - 5
    - Strongly Agree (6)

7. **I support the development of vaccines to prevent STIs.**
    - Strongly Disagree (1)
    - 2
    - 3
    - 4
    - 5
    - Strongly Agree (6)

8. **Vaccines for STIs should be widely available.**
    - Strongly Disagree (1)
    - 2
    - 3
    - 4
    - 5
    - Strongly Agree (6)

9. **I am concerned about the safety of vaccines for STIs.**
    - Strongly Disagree (1)

- 2
- 3
- 4
- 5
- Strongly Agree (6)

10. **The benefits of vaccines for STIs outweigh potential side effects or risks.**
    - Strongly Disagree (1)
    - 2
    - 3
    - 4
    - 5
    - Strongly Agree (6)

11. **Vaccines for STIs should be a part of routine healthcare.**
    - Strongly Disagree (1)
    - 2
    - 3
    - 4
    - 5
    - Strongly Agree (6)

12. **Vaccinating against STIs could lead to risky sexual behaviour.**
    - Strongly Disagree (1)
    - 2
    - 3
    - 4
    - 5
    - Strongly Agree (6)

Attitudes Toward Getting Vaccinated for Gonorrhoea

13. **Before this survey, I knew what gonorrhoea was.**
    - Strongly Disagree (1)
    - 2
    - 3
    - 4
    - 5
    - Strongly Agree (6)
    -

14. **People at risk of infection should be vaccinated against sexually transmitted infections.**
    - Strongly Disagree (1)
    - 2
    - 3
    - 4
    - 5
    - Strongly Agree (6)

15. **I am comfortable getting a vaccine against gonorrhoea**

- Strongly Disagree (1)
- 2
- 3
- 4
- 5
- Strongly Agree (6)

16. **Vaccinating against gonorrhoea could protect from serious health problems in the future.**
    - Strongly Disagree (1)
    - 2
    - 3
    - 4
    - 5
    - Strongly Agree (6)

17. **Vaccinating people against STIs could encourage them to engage in sexual activity**
    - Strongly Disagree (1)
    - 2
    - 3
    - 4
    - 5
    - Strongly Agree (6)

18. **I am more likely to accept a vaccine if the symptoms of the disease it prevents are severe.**
    - Strongly Disagree (1)
    - 2
    - 3
    - 4
    - 5
    - Strongly Agree (6)

19. **I am more likely to accept a vaccine if the disease it prevents cannot be cured.**
    - Strongly Disagree (1)
    - 2
    - 3
    - 4
    - 5
    - Strongly Agree (6)

20. **I think my family would be supportive of me receiving a vaccine against gonorrhoea**
    - Strongly Disagree (1)
    - 2
    - 3
    - 4
    - 5

- Strongly Agree (6)

20. **I think my friends would support me getting vaccinated against gonorrhoea**.
    - Strongly Disagree (1)
    - 2
    - 3
    - 4
    - 5
    - Strongly Agree (6)

Section 3: Attitudes Toward the Potential Gonorrhoea Vaccination Program

This section is to help us understand what you think about a possible program that would offer you a vaccine for gonorrhoea using the men b vaccine. The men b vaccine is already used in babies to protect against meningitis and has shown some protection against gonorrhoea. The idea is to offer this vaccine to people who are at a high risk of bacterial STI infections, or who have had one recently, to try and prevent future infection.
Please mark the choices that are the closest to how you feel. There are no right or wrong responses.

Support for the Gonorrhoea Vaccination Program

1. **I support the introduction of a gonorrhoea vaccination program.**
    - Strongly Disagree (1)
    - 2
    - 3
    - 4
    - 5
    - Strongly Agree (6)

2. **I think that vaccinating just the people at high risk of gonorrhoea infection could be a good way to prevent this STI in the long term.**
    - Strongly Disagree (1)
    - 2
    - 3
    - 4
    - 5
    - Strongly Agree (6)

3. **I think that vaccinating adolescents, before they become sexually active, could be a good way to prevent this STI in the long term.**
    - Strongly Disagree (1)
    - 2
    - 3
    - 4
    - 5

- Strongly Agree (6)

4. **I think that including the gonorrhoea vaccine in the routine vaccination schedule before people become sexually active, and a booster dose when they're older, makes the most sense**
   - Strongly Disagree (1)
   - 2
   - 3
   - 4
   - 5
   - Strongly Agree (6)

5. **I believe that using the meningitis vaccine against gonorrhoea is more acceptable because protection is also given against meningitis**
   - Strongly Disagree (1)
   - 2
   - 3
   - 4
   - 5
   - Strongly Agree (6)
   -

6. **I believe that using the meningitis vaccine against gonorrhoea is more acceptable because babies in the UK have already been given this vaccine.**
   - Strongly Disagree (1)
   - 2
   - 3
   - 4
   - 5
   - Strongly Agree (6)

7. **I believe that using the meningitis vaccine against gonorrhoea is safe because babies in the UK have already been given this vaccine.**
   - Strongly Disagree (1)
   - 2
   - 3
   - 4
   - 5
   - Strongly Agree (6)

Concerns and Considerations

8. **I am worried about the safety of the men b vaccine for preventing gonorrhoea.**
   - Strongly Disagree (1)
   - 2
   - 3
   - 4
   - 5

- Strongly Agree (6)

9. **I am worried about the efficacy (how well it works) of the men b vaccine for preventing gonorrhoea.**
   - Strongly Disagree (1)
   - 2
   - 3
   - 4
   - 5
   - Strongly Agree (6)

10. **I would like more information on how well the vaccine works against gonorrhoea long-term before supporting its use**
    - Strongly Disagree (1)
    - 2
    - 3
    - 4
    - 5
    - Strongly Agree (6)

11. **I think the lower level of protection given by the MenB vaccine against gonorrhoea is outweighed by the protection also given against meningitis**
    - Strongly Disagree (1)
    - 2
    - 3
    - 4
    - 5
    - Strongly Agree (6)
12. **I think the lower level of protection given by the MenB vaccine against gonorrhoea is outweighed by the large amount of safety data, and that the vaccine is already given to babies in the UK**
    - Strongly Disagree (1)
    - 2
    - 3
    - 4
    - 5
    - Strongly Agree (6)

13. **I am worried that introducing a gonorrhoea vaccine might cause people to take part in risky sexual behaviour.**
    - Strongly Disagree (1)
    - 2
    - 3
    - 4
    - 5
    - Strongly Agree (6)

14. **I believe that there should be an educational campaign about the benefits and risks of the gonorrhoea vaccine.**
    - Strongly Disagree (1)
    - 2
    - 3
    - 4
    - 5
    - Strongly Agree (6)

15. **I believe that there should be a focus on education to prevent STIs (safe sex, use of condoms etc) rather than a vaccine programme**
    - Strongly Disagree (1)
    - 2
    - 3
    - 4
    - 5
    - Strongly Agree (6)

Implementation and Accessibility

16. **How important is convenience in deciding whether to get vaccinated?**
    - Extremely important
    - Very important
    - Moderately important
    - Slightly important
    - Not important

17. **How would you prefer to book an appointment for a vaccine?** (Check all that apply)
    - Online booking system (via website/app)
    - Walk-in without an appointment
    - By phone call
    - Through a healthcare provider referral
    - Other (please specify)

18. **What are the most convenient times for you to schedule a vaccine appointment?** (Check all that apply)
    - Morning (8 am - 12 pm)
    - Afternoon (12 pm - 4 pm)
    - Evening (4 pm - 8 pm)
    - Weekends
    - Anytime during the day

19. **How far would you be willing to travel to get vaccinated?**
    - Less than 5 minutes
    - 5-10 minutes
    - 10-20 minutes
    - 20-30 minutes
    - Over 30 minutes

20. **Where would you prefer to get vaccinated?** (Check all that apply)
    - Pharmacy
    - GP surgery
    - Sexual health clinic
    - Hospital
    - University/College campus
    - Pop-up vaccination site (e.g., shopping centre, community centre)
    - Drive-through vaccination site
    - Other (please specify)

21. **What would make it easier for you to access a vaccination site?** (Check all that apply)
    - Easy public transportation options
    - Free transportation services (e.g., bus pass, taxi voucher etc)
    - Short waiting times
    - Flexible appointment times (e.g., late evenings, weekends)
    - Availability of walk-in appointments
    - Clear information on location and availability
    - Other (please specify)

22. **How important is it that the cost of the gonorrhoea vaccine should be covered by public health services to ensure everyone who wants it can get it?**
    - Extremely important
    - Very important
    - Moderately important
    - Slightly important
    - Not important

Section 4: Vaccine Communication and Education
This section aims to understand where you get your information about vaccines and STIs and how much you trust these sources (click all that are relevant).
.

Education Requirements

1. **What type of educational content do you think is necessary for you about the potential gonorrhoea vaccination program?**
    - Basic information about the men b vaccine
    - Details about gonorrhoea (cases, symptoms, long-term effects)
    - Information about the effectiveness and safety of the vaccine
    - Explanation of how the vaccine fits into the existing vaccination schedule
    - The importance of vaccination for STI prevention
    - Other (please specify): ___________

Who Should Deliver the Education?

3. **Who do you believe is the most suitable for delivering educational content about the gonorrhoea vaccination to you?**
    - Healthcare workers (e.g., doctors, nurses)
    - School teachers, university lecturers, or counsellors

- Peer educators (e.g., other students)
- Public health officials
- Online resources (e.g., websites, social media)
- Parents or caregivers
- Other (please specify): ___________

Message Framing

5. **How should the message about the gonorrhoea vaccination be explained to make it most effective?**
    - Emphasise the dual protection against meningitis and gonorrhoea
    - Focus on the prevention of gonorrhoea specifically
    - Highlight the benefits of including it in the routine vaccination schedule
    - Stress the importance of preventing STIs as part of overall health
    - Stress the importance of preventing STIs as part of public health (health of everyone in the country)
    - Stress the importance of preventing STIs as part of global health (health of everyone in the world)
    - Other (please specify): ___________

6. **Which aspects of the vaccine should be emphasised in communications to ensure acceptance and understanding?**
    - The safety and efficacy of the men b vaccine
    - The potential long-term benefits of preventing gonorrhoea
    - Information on the current rates of gonorrhoea and antimicrobial resistance
    - The integration of the vaccine into the existing vaccination schedule
    - Success stories or evidence from similar vaccination programs
    - Other (please specify): ___________

Information to Provide

7. **What specific information about gonorrhoea should be included in educational materials?**
    - Current number of gonorrhoea cases
    - Symptoms and health consequences of gonorrhoea
    - Information on antimicrobial resistance and treatment challenges
    - How gonorrhoea is transmitted and prevented
    - The benefits of early vaccination and its impact on public health
    - The impact of gonorrhoea on health in developing countries
    - Other (please specify): ___________

8. **What specific information about the men b vaccine should be included?**
    - How the vaccine works and how well it works
    - Potential side effects and safety
    - The process and schedule for vaccination
    - Any known interactions with other vaccines
    - Comparisons to other STI prevention methods
    - Other (please specify): ___________

Delivery Methods

8. **What methods would be most effective for delivering educational content to you?**
    - School/college/university-based programs and presentations
    - Peer-led education sessions
    - Online resources and interactive tools
    - Educational videos and social media campaigns
    - Health education classes or modules
    - Other (please specify): ___________

Current information sources

9. **If you wanted to get information about vaccines and STIs, where would you get this from? (Select all that apply)**
    - School/College/University
    - Parents/Guardians
    - Friends
    - Social Media
    - Health Professionals (e.g., doctors, nurses)
    - Websites
    - Other (please specify):
    - Prefer not to say

10. **How much do you trust the following sources for information about vaccines and STIs?**

    - School/College/Universities
        1. Not at all
        2. A little
        3. Moderately
        4. Very much
    - Parents/Guardians
        1. Not at all
        2. A little
        3. Moderately
        4. Very much

    - Friends
        1. Not at all
        2. A little
        3. Moderately
        4. Very much

    - Social Media
        1. Not at all
        2. A little
        3. Moderately
        4. Very much

    - Health Professionals (e.g., doctors, nurses)
        1. Not at all

2. A little
3. Moderately
4. Very much

- Websites
    1. Not at all
    2. A little
    3. Moderately
    4. Very much

11. How easy do you find it to get information about vaccines or STIs from each of the following sources?

- School/College/Universities
    1. Not at all easy
    2. Slightly easy
    3. Moderately easy
    4. Very easy

- Parents/Guardians
    1. Not at all easy
    2. Slightly easy
    3. Moderately easy
    4. Very easy

- Friends
    1. Not at all easy
    2. Slightly easy
    3. Moderately easy
    4. Very easy

- Social Media
    1. Not at all easy
    2. Slightly easy
    3. Moderately easy
    4. Very easy

- Health Professionals (e.g., doctors, nurses)
    1. Not at all easy
    2. Slightly easy
    3. Moderately easy
    4. Very easy

- Websites
    1. Not at all easy
    2. Slightly easy
    3. Moderately easy
    4. Very easy

Section 5: Final Thoughts

1. Do you have any additional comments or thoughts about gonorrhoea vaccination for young adults?

Thank you for taking part in our survey – we really appreciate it.

Once we have analysed all the data, our findings will be available on www.ovg.ox.ac.uk

We will also be conducting semi-structured interviews to explore the topics covered in this survey in more depth. We will be conducting c20 interviews. If you would like to be contacted to potentially take part in an online interview, please include your email address below.
