# Supplementary material for: “I would walk through fire to get this vaccine”: a mixed-methods study examining attitudes and perceptions of a gonorrhoea vaccine programme among UK sexual health service users
Source: BMJ Public Health. 2026 Mar 27;4(1):e003819. doi: 10.1136/bmjph-2025-003819 (PMC13034242; doi:10.1136/bmjph-2025-003819)
Supplement: online supplemental file 3 [file bmjph-4-1-s003.pdf]

NAVIGATE: Navigating Attitudes to Vaccination Implementation for Gonorrhoea in the UK: Acceptability, Trust and Engagement

Sexual Health Service User Interview Guide

Introduction and Consent Confirmation

- Briefly introduce the purpose of the interview and reassure confidentiality and take consent for audio recording. " Can you please confirm you are happy for me to record our conversation using an external audio recorder? I will then make a note of this consent, and send you the completed consent form after our conversation".
- Thank you for taking part in this interview about your views on a potential vaccination program for adolescents to prevent gonorrhoea.

Section 1: General Attitudes Toward Vaccination (VAX)

General Vaccination Perceptions

- Can you share your general thoughts about vaccines?
- How do you feel about the role of vaccines in preventing diseases?
- Do you trust vaccines to protect against serious illnesses?

Section 2: Attitudes Toward Sexual Health Vaccination

STI Perceptions

- How serious do you think sexually transmitted infections (STIs) are for individuals and the community as a whole?
- How do you feel about the current methods available for preventing STIs? Are they effective enough?

STI Vaccine Acceptance

- If vaccines were available to prevent STIs, how supportive would you be of making them part of routine healthcare?
- What are your thoughts on the safety and effectiveness of STI vaccines in general?

Gonorrhoea Vaccine-Specific Questions

- How comfortable or willing would you be to get a vaccine specifically for gonorrhoea?
- How do you perceive the severity of gonorrhoea symptoms and its resistance to treatment? Does this affect your willingness to get vaccinated?
- Would the opinions of family and friends influence your decision to get a gonorrhoea vaccine?

Section 3: Attitudes Toward a Potential Gonorrhoea Vaccination Program

General Support for a Gonorrhoea Vaccination Program

- Would you generally support a gonorrhoea vaccination program, particularly for high-risk individuals or adolescents?

Concerns About a Gonorrhoea Vaccine Program

- Do you have any thoughts regarding the safety or effectiveness of a gonorrhoea vaccine?

- How would you feel about taking a vaccine that offers less than 50% protection?
- Do you think that the acceptability of taking a vaccine that offers lower levels of protection against gonorrhoea is higher when the vaccine also offers protection against another disease too? Why/why not?
- What are your thoughts on the use of a low efficacy vaccine if it is already licensed and used in babies, and therefore there are data available regarding the safety in a general population?
- Do you think that having a gonorrhoea vaccine available might encourage risky sexual behaviours? Why or why not?

Section 5: Implementation and Accessibility
Convenience and Accessibility
- How important is it for you that getting vaccinated is convenient (e.g., easy booking, location accessibility)?

Appointment Preferences
- How would you prefer to book an appointment for a vaccination: online, walk-in, phone, or referral?

Preferred Locations
- Where would you feel most comfortable getting a gonorrhoea vaccine? (Pharmacies, GP surgeries, sexual health clinics, etc.)

Cost Coverage
- How important is it to you that the cost of the gonorrhoea vaccine is covered by public health services?
- Have you ever received private health services? Would you pay for a gonorrhoea vaccine?

Section 6: Vaccine Communication and Education
Education and Information Needs
- What type of information would you need to feel informed about a gonorrhoea vaccine (e.g., details about the vaccine, facts about gonorrhoea)?

Preferred Educators
- Who would you trust most to deliver information about the vaccine? (Healthcare workers, teachers, peer educators, online resources)

Message Framing
- What kind of messages would you find most persuasive about getting a gonorrhoea vaccine? (e.g., dual protection against gonorrhoea and meningitis, routine healthcare inclusion, impact on global health)

Delivery Methods
- How should education about a gonorrhoea vaccine be delivered? (prompts: school programs, peer-led sessions, online tools, social media)

Section 7: Current Information Sources
Sources of Information
- Where do you currently get your information about sexual health and vaccines? (School, parents, social media, health professionals, websites)

Trust in Information Sources

- Which sources of information do you trust most? (School, parents, friends, social media) Why?

Closing Remarks

- Thank the participant for their time and input.
- Ask if they have any final thoughts or questions about gonorrhoea vaccination.
- Remind them they can reach out if they want to discuss anything further or access more information.
