# Supplementary material for: “I would walk through fire to get this vaccine”: a mixed-methods study examining attitudes and perceptions of a gonorrhoea vaccine programme among UK sexual health service users
Source: BMJ Public Health. 2026 Mar 27;4(1):e003819. doi: 10.1136/bmjph-2025-003819 (PMC13034242; doi:10.1136/bmjph-2025-003819)
Supplement: online supplemental file 4 [file bmjph-4-1-s004.pdf]

Supplementary material 4 – Summary of qualitative findings

Theme	Subtheme	Summary of finding	Illustrative quotation
Programme justification	Antimicrobial resistance (AMR)	Rising AMR framed vaccination as necessary and urgent	"Whatever we can do to cut down the spread… is probably the most sensible thing." (Male, 45–54)
Programme justification	Limits of behavioural prevention	Condoms and PrEP seen as adherence-dependent, positioning vaccination as complementary	"They're very user dependent… it's all event based." (Male, 25–34)
Trust and confidence	Institutional trust and safety	Confidence in UK regulation and prior MenB use supported acceptability	"They wouldn't be approved as a vaccine in the UK otherwise." (Male, 25–34)
Trust and confidence	Transparency and effectiveness	Participants favoured clear, realistic information about partial effectiveness	"40% is better than zero." (Male, 35–44)
Access and stigma	Preferred delivery settings	Sexual health clinics perceived as less stigmatising than general healthcare	"Any GUM clinic would work… not my local pharmacy." (Male, 35–44)
Access and equity	Cost as a barrier	Even modest costs perceived as worsening inequalities	"Someone on 21–24k a year… every penny counts." (Male, 35–44)
Communication	Peer-led and community messaging	Trusted peers and LGBTQ+ figures viewed as central to effective dissemination	"I would definitely trust fellow gays… gay influencers." (Male, 35–44)
Communication	Careful messaging	Sensitivity around messaging to avoid inducing fear or overpromising	"You'd need to avoid a confusing message where people believe… they're some kind of Superhuman who's never going to catch either" (Male, 35-44).
Communication	Dual benefit framing	The potential for dual protection, against both MenB and gonorrhoea, was received positively when framed as an unexpected advantage	"It's like… opening your Cornetto ice cream box where they say… it's five and you find the sixth one" (Male, 35-44)."
