# Supplementary material for: “I would walk through fire to get this vaccine”: a mixed-methods study examining attitudes and perceptions of a gonorrhoea vaccine programme among UK sexual health service users
Source: BMJ Public Health. 2026 Mar 27;4(1):e003819. doi: 10.1136/bmjph-2025-003819 (PMC13034242; doi:10.1136/bmjph-2025-003819)
Supplement: online supplemental file 5 [file bmjph-4-1-s005.pdf]

CROSS Checklist – Survey Component of Mixed-Methods Study

This checklist was completed for the survey component of the mixed-methods study titled: “I would walk through fire to get this vaccine”: Attitudes and perceptions of a gonorrhoea vaccine programme among UK sexual health service users.

Note: This checklist addresses only the survey portion. The qualitative interview data is excluded from this reporting checklist. The full survey instrument is provided as Supplementary Material 2.

Section / Item	Description	Addressed?	Details / Location
Title & Abstract			
1. Title	Identify the study as a survey in the title.	n/a	Not exclusively a survey study; abstract specifies survey.
2. Abstract	Provide structured summary of objectives, methods, results, and conclusions.	Yes	Abstract contains survey design, methods, and findings.(page 2-3)
Introduction			
3. Background	Scientific background and rationale.	Yes	Addressed in introduction. (page 6-8)
4. Objectives	Specific objectives and/or research questions.	Yes	Stated clearly in introduction. (page 8)
Methods			
5. Study Design	Describe the study design.	Yes	Mixed-methods study; survey design described. (page 8)
6. Setting	Describe the setting and locations.	Yes	Online survey, social media and clinic recruitment.(page 10-11)
7. Participants	Eligibility criteria and selection method.	Yes	Adults, UK residents, past use of sexual health services. (page 8)
8. Variables	Define variables and survey domains.	Yes	Demographics, VAX scale, attitudes.(page 10-11)
9. Data Sources / Measurement	Survey instruments and measurement.	Yes	VAX scale cited; structure described.(page 10-11)

10. Bias	Potential sources of bias.	Yes	Acknowledged in Limitations section.(page 31)
11. Study Size	How sample size was determined.	Yes	Calculation and rationale described.(page 9)
12. Quantitative Variables	Handling of variables in analysis.	Yes	Descriptive analysis in SPSS. (page 11-12)
13. Statistical Methods	Describe analysis methods.	Yes	Descriptive statistics; qualitative excluded here. (page 11-12)
Results			
14. Participants	Number at each stage of the survey.	Yes	500 started; 395 completed. (page 13)
15. Descriptive Data	Characteristics of participants.	Yes	Tables 1 and 2 provided. (page 14-16)
16. Outcome Data	Main findings related to objectives.	Yes	VAX results and attitudes shown. (page17-20)
17. Main Results	Key results with CIs if applicable.	Yes	Percentages reported; no inferential stats. (page17-20)
Discussion			
18. Key Results	Summarise findings in relation to objectives.	Yes	Summarised in Discussion. (page 27-32)
19. Limitations	Discuss limitations and bias.	Yes	Bias and generalisability addressed. (page 31-32)
20. Interpretation	Interpret findings given limitations.	Yes	Well integrated with past literature. (page 27-31)
21. Generalisability	Discuss external validity.	Yes	Sample is powered but limited generalisability; discussed. (page 31)
Other Information			
22. Ethics	Ethical approval and consent.	Yes	Approval cited (MSD IDREC 947232). (page 12)
23. Funding	Funding and role of funders.	Yes	Funders had no role; disclosed. (page 33)

24. Conflicts of Interest	Disclosure of competing interests.	Yes	Disclosed in manuscript and COI forms (page 34)
25. Survey Instrument	Provide full survey.	Ye	Summary in text; full version as Supplementary Material 2.

Sharma A, Minh Duc NT, Luu Lam Thang T, Nam NH, Ng SJ, Abbas KS, Huy NT, Marušić A, Paul CL, Kwok J, Karbwang J, de Waure C, Drummond FJ, Kizawa Y, Taal E, Vermeulen J, Lee GHM, Gyedu A, To KG, Verra ML, Jacqz-Aigrain ÉM, Leclercq WKG, Salminen ST, Sherbourne CD, Mintzes B, Lozano S, Tran US, Matsui M, Karamouzian M. A Consensus-Based Checklist for Reporting of Survey Studies (CROSS). J Gen Intern Med. 2021 Oct;36(10):3179-3187. doi: 10.1007/s11606-021-06737-1. Epub 2021 Apr 22. PMID: 33886027; PMCID: PMC8481359.

SRQR Checklist – Qualitative Component of Mixed-Methods Study

This checklist corresponds to the qualitative component of the mixed-methods study titled:
“I would walk through fire to get this vaccine”: Attitudes and perceptions of a gonorrhoea vaccine programme among UK sexual health service users.

Note: This SRQR (Standards for Reporting Qualitative Research) checklist covers the interview-based qualitative findings reported in the manuscript.

Item No.	SRQR Item Description	Reported?	Details / Manuscript Location
1	Concise description of the nature and topic of the study; identification as qualitative.	n/a	Study was not purely qualitative; abstract and methods specify semi-structured interviews.
2	Summary including background, purpose, methods, results, and conclusions.	Yes	Abstract summarizes qualitative purpose, design, and key insights. (page 2-3)
3	Description and significance of the problem studied; review of relevant theory and empirical work.	Yes	Framed in the Introduction with prior literature and rationale for qualitative exploration. (page 6-8)
4	Purpose of the study and specific objectives or questions.	Yes	Outlined in Introduction. (page 8)
5	Qualitative approach and guiding theory or paradigm.	Yes	This approach was grounded in a pragmatic paradigm, enabling the integration of quantitative and qualitative data to comprehensively explore both the prevalence and contextual drivers of vaccine attitudes. (page 8)

6	Researcher attributes, relationship with participants, assumptions, etc.	Partially	Not explicitly described as limited by word count, though the research team and their reflexivity was discussed, alongside public contributors. (page 8)
7	Setting/site and salient contextual factors.	Yes	Participants were UK sexual health service users; context discussed throughout. (page 9)
8	How and why participants were selected; criteria for saturation.	Yes	Described in Sampling section; thematic saturation targeted with purposive sampling. (page 9-10)
9	Ethics approval and participant consent.	Yes	Ethical approval and consent process detailed in Ethics section. (page 12)
10	Types of data collected, procedures, and rationale.	Yes	Semi-structured interviews; procedures and tools described. (page 9-12)
11	Instruments (interview guide) and devices used.	Yes	Interview guide co-designed with public contributors; interviews recorded and transcribed. (Guide attached as supplement material 3)
12	Number and relevant characteristics of participants.	Yes	Demographic table (Table 1 and 2) provided. (page 14-16)
13	How data were handled, coded, and anonymized.	Yes	Verbatim transcription and anonymisation described. (page 10-12)
14	Process for identifying themes or patterns,	Yes	Braun and Clarke's thematic analysis framework used;

	including researchers involved.		collaborative coding described. (page 2)
15	Techniques such as triangulation, member checking, audit trails.	Yes	Triangulation with survey data described; iterative coding framework development. (page 12)
16	Main findings and their derivation.	Yes	Themes presented in Results with supporting quotes. (page 13-27)
17	Evidence (quotes) to substantiate themes.	Yes	Rich participant quotes used throughout.(page13-27)
18	Findings related to existing literature and theory.	Yes	Discussion integrates findings with broader literature on AMR, stigma, etc. (page 27-33)
19	Trustworthiness and limitations of findings.	Yes	Limitations of sample and generalisability discussed. (page 31-32)
20	Potential sources of influence or bias.	Yes	Conflicts disclosed under 'Competing interests'. (page 34)
21	Sources of funding and role of funders.	Yes	Funding described; funders had no role in analysis or writing. (page 33)

O'Brien BC, Harris IB, Beckman TJ, Reed DA, Cook DA. Standards for reporting qualitative research: a synthesis of recommendations. Acad Med. 2014;89(9):1245-1251.

GRAMMS Checklist – Good Reporting of a Mixed Methods Study

Checklist Item	Addressed in Manuscript?	Location/Notes
Justification: Explain why a mixed methods approach was necessary	Yes	Quantitative results were used to inform and contextualise qualitative insights, and vice versa, enabling a more comprehensive understanding of participants' attitudes and perceptions. (page 12)
Design: Describe the mixed methods design and rationale	Yes	Page 8-12 – methods section
Integration: Describe how quantitative and qualitative data were integrated	Yes	Page 8-12 – methods section
Interpretation: Describe how integration influenced interpretation	Yes	This integration enabled interpretation of survey findings through the lens of lived experience, revealing how structural and social barriers shaped otherwise high levels of stated vaccine acceptability. (page 12)
Limitations: Discuss limitations specific to the mixed methods design	Yes	First, a limitation of the mixed-methods design is that while the qualitative data added valuable depth to interpretation, it was collected from a smaller and more demographically homogeneous subset of survey participants, which may limit representativeness. (page 31)

O'Cathain A, Murphy E, Nicholl J. The quality of mixed methods studies in health services research. J Health Serv Res Policy. 2008;13(2):92-98.
